# Hope for better days: 2 studies aiming to answer the remaining questions around dual mobility cups

**DOI:** 10.1080/17453674.2020.1817663

**Published:** 2020-09-10

**Authors:** Jan Verhaar

**Affiliations:** Department of Orthopaedics and Sports Medicine, Erasmus University Medical Center, Rotterdam, The Netherlands

Readers of scientific papers are mostly interested in new findings. By scanning the paper’s title, reading the summary and, when attracted by the conclusions, reading the full article, we hope to find new information that will help us to treat our patients better. In this issue of Acta Orthopaedica there are 2 excellent papers, which I would recommend reading, but I need to warn readers beforehand. They do not contain new data yet, but are still worth the time investment required to read them (Van Beers et al. 2020, Wolf et al. 2020).

Both articles concern the value of dual mobility cups (DMC). DMCs came onto the market to reduce the risk of dislocation after total hip arthroplasty. A spherical liner in a DMC encloses the metal femoral head and articulates with a thin metal shell, which is fixed to the acetabular bone. The first DMCs were developed in France, and publications from French colleagues suggested better range of motion and reduced dislocation rates. In many countries the enticing concept of DMC was adopted, especially in patients with high risk of dislocation, and in many patients after revision hip surgery. Based on observational studies, the concept seems to work by reducing dislocation risks. But there are no well-powered randomized trials showing this clearly and there are still safety concerns because of the large polyethylene liner in a metal shell. Wear, aseptic loosening, periprosthetic infection, and intraprosthetic dislocation are all potential complications not yet studied in great detail.

Due to the pioneering work of Scandinavian orthopedics, there are many well-run national implant registers worldwide, but unfortunately they cannot provide all the answers to the above questions. Implant registers report only dislocations that lead to revisions, but without surgery there is no information on the actual dislocation rate. The only solution for the problem is a proper randomized controlled trial (RCT) and both papers give detailed descriptions of such study designs.

Wolf et al. (2020) aim to perform a national (Swedish), multicenter, register-based RCT comparing a DMC with a standard cup in patients > 65 years with a non-pathological, displaced femoral neck fracture. Van Beers et al. (2020) plan to perform a similar national (Dutch) RCT but they compare a DMC with normal cup in all patients ≥ 70 years undergoing elective primary hip arthroplasty. The power calculation of both studies leads to quite a high number of patients for each study—1,100 in the Dutch study and 1,600 in the Swedish study—and this explains why multiple centers are needed, to include sufficient patients.

The papers illustrate excellently how much energy the preparation of such studies requires from the researchers. However, not only the research plan but also the weakness of the studies are already openly and thoroughly considered in the Discussion section of the respective articles. This shows that even the best research plan is still a compromise and several good studies are necessary to reach the right conclusions.

A real challenge for all studies of this size is the follow-up. How to keep track of all the patients? For this reason, both studies are nested in the national arthroplasty registries (the Swedish Hip Arthroplasty Register and the Dutch Arthroplasty Register). After final study follow-up, all participants remain traceable in the arthroplasty registers for evaluation of long-term survival and mortality. Both studies will trace complications leading to further surgery. The Swedish study will use the Swedish National Patient Register to detect all dislocations, not only those leading to surgery. The researchers of the Swedish study have a clear advantage over the Dutch study, which intends to detect these dislocations with a questionnaire sent at 3-month, 1-year, and 2-year follow-up. Importantly, both studies also include health-economic evaluation of the use of dual mobility cups, which is relevant for society. Will the increased cost associated with DMC reduce total costs, including those caused by dislocations?

The weakness of many orthopedic interventions is that their scientific foundation is weak and the complications and healthcare economic consequences are insufficiently studied. dual mobility cups are a clear example. They are promising but the proof is doubtful. Based on ambition and using the best scientific tools as well as the excellent options from the national arthroplasty registers, the planned studies from Sweden and the Netherlands give us hope of a more knowledgeable future.

Jan Verhaar *Department of Orthopaedics and Sports Medicine* *Erasmus University Medical Center* *Rotterdam* *The Netherlands* *email:*j.verhaar@erasmusmc.nl
